# Translational benefits to patients in the post-genomic era

**DOI:** 10.1186/2046-2530-1-S1-K1

**Published:** 2012-11-16

**Authors:** JG Gleeson

**Affiliations:** 1 Howard Hughes Medical Institute (HHMI), Department of Neurosciences, University of California, San Diego, USA

## 

The transformative discoveries in the field of genetics have led to a revolution in the way that diseases of genetic origin can be studied and treated. The ability to profile a patient’s genome to uncover all of the genetic variants at once now provides physicians the opportunity to better understand each patient’s disease and provides scientists a new window into pathogenesis. The translation of ‘next generation’ sequencing directly to the clinic is still being assessed, but holds great promise for genetic disease to reduce costs, advance accuracy, and point to unsuspected and treatable conditions. To study its capability in the clinic, we performed whole exome sequencing (WES) in 118 probands with a diagnosis of a pediatric-onset neurodevelopmental disease in which most known causes had been excluded. Among several novel genes identified in this study were *EXOC8* in Joubert syndrome and *GFM2* in microcephaly, simplified gyral pattern, and insulin-dependent diabetes, further establishing WES as a useful tool for novel gene discovery. Importantly, exome sequencing uncovered 10 probands with mutations in genes known to cause a disease different from the initial diagnosis. Upon further medical evaluation, these mutations were found to account for each proband’s disease, leading to a change in diagnosis, some of which led to changes in management. This data provide proof-of-principle that genomic strategies are useful in reclassifying diagnosis in a substantial proportion of patients. In order to explore potential treatments for ciliopathy disorders, we devised a cell-based screen, using a pharmacologically-relevant RNAi library to search for genes modulating ciliogenesis and cilia stability. Among the hits were several genes in the actin regulatory network. We found that genes that promote actin stability led to an inhibition of ciliogenesis, whereas genes that inhibit actin stability promoted ciliogenesis. Cytochalasin, a drug with potent actin inhibitory activity, promoted ciliogenesis both in wildtype cells as well as cells with mutations in key ciliogenesis gene. While cytochalasin itself might be too toxic for human use, these findings suggest pharmacological approaches to treating ciliopathies. We have extended these findings by probing the connection between ciliogenesis and the cell cycle and have uncovered a node centered around *HNF4*, encoding Hepatocyte nuclear factor 4, as a module linking these two cellular stages. We are interested in optimizing high-content cell-based screening strategies to identify functional genetic requirements for a host of important cell biological questions in the field of cilia in development and disease.

